# First European Clinical Implant of an Off-the-Shelf Bioengineered Blood Vessel for Coronary Artery Bypass

**DOI:** 10.3390/jcm15083003

**Published:** 2026-04-15

**Authors:** Mateusz Kuć, Matthew Soule, Zeeshan Syedain, Abrielle Krouse, Łukasz Wójcik, Monika Chomej-Dąbrowska, Patryk Król, Jerzy Pacholewicz

**Affiliations:** 1 Department of Cardiac Surgery, Pomeranian Medical University, 70-111 Szczecin, Poland; 2 Department of Surgery, University of Minnesota Medical School, Minneapolis, MN 55455, USA; 3 Department of Biomedical Engineering, University of Minnesota, Minneapolis, MN 55455, USA; 4 Vascudyne, Saint Paul, MN 55017, USA; 5 Department of Radiology, Pomeranian Medical University, 70-111 Szczecin, Poland; 6 Clinical Trials Support Center, Pomeranian Medical University, 71-252 Szczecin, Poland

**Keywords:** coronary artery bypass grafting, tissue-engineered vascular graft, external stenting, bioengineered conduit, case report

## Abstract

**Background**: Coronary artery bypass grafting is the optimal revascularization strategy for patients with complex multivessel coronary artery disease. However, saphenous vein grafts are associated with high failure rates and donor site morbidity. Off-the-shelf tissue-engineered vascular grafts offer a potential solution for patients lacking suitable autologous vessels. Here, we report the first successful clinical implant of an acellular Tissue-Engineered Vessel (TEV) for coronary artery bypass grafting in Europe. **Methods**: A 73-year-old male with two-vessel disease and no suitable autologous vein underwent on-pump coronary artery bypass grafting using the left internal mammary artery to the left anterior descending artery and a 4 mm TEV to the right coronary artery. **Results**: Implant procedure followed standard surgical techniques, sutures and duration. The conduit handling was comparable to native vessels. Intraoperative flow measurements demonstrated excellent graft performance (TEV: 110 mL/min, Pulsatility Index 1.0). Postoperative recovery was uneventful. One-month computed tomography coronary angiography confirmed graft patency. **Discussion**: This case demonstrates the feasibility of using a bioengineered conduit for coronary revascularization in patients without suitable autologous grafts. If these findings are confirmed in larger trials, bioengineered vessels could expand surgical revascularization to patients without suitable autologous conduits and fundamentally alter conduit selection strategy in CABG. **Conclusions**: This first-in-Europe clinical implant demonstrates that an off-the-shelf acellular tissue-engineered vessel can meet the procedural, hemodynamics, and patency requirements of coronary artery bypass. These proof-of-concept results support progression to prospective multi-center evaluation.

## 1. Introduction

Coronary artery disease (CAD) continues to be a leading cause of morbidity and death globally, with many cases requiring surgical treatment [1]. For individuals with complex multivessel disease, coronary artery bypass grafting (CABG) remains the standard of care, offering improved survival rates and better quality of life [2,3]. This procedure typically involves utilizing segments of healthy autologous blood vessels, typically the internal mammary artery (IMA), saphenous vein grafts (SVG), or radial artery (RA), to bypass narrowed or blocked coronary arteries [4,5].

Although SVGs are frequently chosen due to their accessibility and length, saphenous vein grafts (SVG) have significant limitations. Clinical study data has demonstrated that up to 45% of saphenous vein grafts lose patency within one year [4]. Contributing factors include intimal hyperplasia, rapid progression of atherosclerosis, and thrombosis [6,7]. Additionally, many patients have underlying conditions such as peripheral vascular disease, prior vein harvesting, or venous insufficiency which can compromise the quality of available autologous veins or make them unusable altogether [8].

Using compromised veins as bypass conduits can lead to graft failure resulting in major adverse cardiac events including myocardial infarction, recurrent angina, and even death. Furthermore, harvesting autologous vessels introduce secondary surgical sites that can lead to complications such as infection, pain, and delayed wound healing [9,10]. With an increasingly aging patient population and higher risk profiles, there is a growing need for alternative bypass conduits that offer reliable long-term patency while eliminating the complications associated with harvesting autologous vessels.

**Figure 1:**
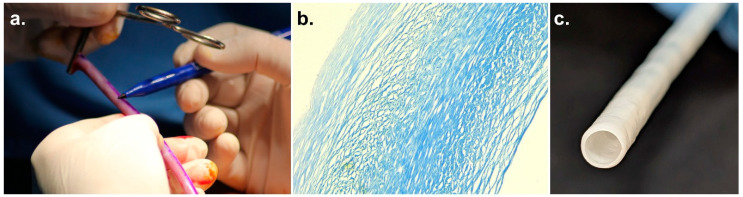
(**a**). Photograph of the TEV during preparation, (**b**). Trichrome image of TEV structure and (**c**). End view of the 4 mm TEV.

Innovations in tissue engineering have introduced promising approaches for developing off-the-shelf vascular conduits that could potentially address these challenges. Once implanted, a tissue-engineered vessel is expected to endothelialize and regenerate to provide a living blood vessel for long-term patency, an outcome not achievable with previous synthetic conduits. Vascudyne has developed the Tissue Engineered Vessel (TEV) (Vascudyne, Saint Paul, MN, USA) by seeding human fibroblasts into a biodegradable fibrin gel scaffold on a tubular mandrel and culturing in a bioreactor. During the bioreactor culture, the cells remodel the fibrin scaffold into a dense, collagen-rich extracellular matrix tube. In the final step, the cellular components are removed. The process can be adapted to multiple diameters, with the 6 mm diameter conduit demonstrating promising results in preclinical models including endothelialization and regeneration [11,12,13]. The 6 mm diameter conduit has also shown safety as a peripheral vascular graft in clinical trials [14,15,16,17,18,19]. Preclinical studies of the TEV in the diameter of 4 mm demonstrated long-term patency in CABG preclinical models out to 18 months [20]. The Vascudyne TEV conduit is under clinical trials and is not approved for commercial use (Figure 1).

This case represents the first clinical proof-of-concept for off-the-shelf bioengineered coronary bypass grafting in Europe, demonstrating that an acellular tissue-engineered vessel can meet the practical demands of coronary revascularization: standard surgical technique, comparable anastomosis time, favorable intraoperative hemodynamics, and confirmed early patency. Beyond technical feasibility, these findings carry broader clinical implications. The availability of a quality-controlled, off-the-shelf conduit could expand the eligibility for surgical revascularization to patients currently deemed suboptimal candidates due to poor quality of native conduits or their unavailability, a population that is growing as patients present with increasingly complex comorbidity profiles. If efficacy is confirmed in larger trials, the TEV could redefine conduit selection strategy in CABG, functioning not merely as a last-resort alternative but as a primary option in selected clinical scenarios.

## 2. Methods

Ethical approval for this study was obtained from the Ethics Committee of the Regional Medical Chamber in Warsaw, and written consent was obtained from this patient.

The patient, a 73-year-old male with two-vessel coronary artery disease (Table 1), was selected for the Vascudyne TEV conduit due to the unavailability of suitable autologous grafts, specifically SVG. Preoperative assessment included coronary angiography, which revealed stenosis in the left anterior descending (LAD) artery and the right coronary artery (RCA). It was determined that the diameter of the distal anastomosis target site on the RCA was at least 1.5 mm and the length of conduit required would be 15 cm or less. The patient met the Inclusion and Exclusion criteria for the trial (NCT07078370). Therefore, the decision was made to use the Vascudyne TEV as the bypass conduit to the RCA.

## 3. Results

**Figure 2:**
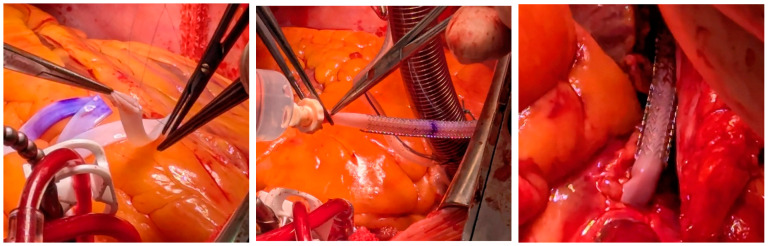
Photographs of the TEV implant, suturing of distal anastomosis, loading of ESS, and proximal anastomosis.

The operation was conducted as an on-pump coronary artery bypass procedure. The left internal mammary artery (LIMA) was harvested and the Vascudyne TEV was prepped. The TEV was implanted, with the distal anastomosis done first, using a 7-0 Corolene suture (Figure 2). After the LIMA to LAD anastomosis, the conduit length was measured and the appropriate Vascudyne ESS was selected. The final 4 mm diameter TEV length was measured to be 14 cm. An ESS-12 (12 cm long, internal diameter 5.4 mm) was prepped and loaded onto the Vascudyne TEV. The proximal anastomosis was completed using a 6-0 Corolene. The patient was separated from cardiopulmonary bypass and protamine administered. Transit time flow measurement was performed in both the TEV and the LIMA before closing (Table 2).

The postoperative course was uneventful, and the patient was discharged from the cardiovascular ward and moved to the rehabilitation unit with aspirin (75 mg daily) and apixaban (5 mg twice daily), which started postoperative Day 6.

**Figure 3:**
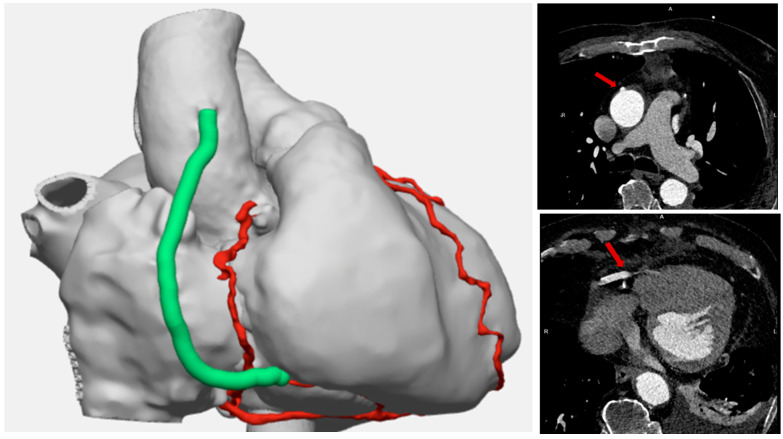
3D model rendering of the proximal and distal anastomosis (green conduit). CT scan showing the proximal and distal anastomosis (red arrows).

One-month computed tomography coronary angiography (CCTA) demonstrated patent conduit (Figure 3). This patient will continue to be followed up at 6 months with CCTA and an angiogram at 12 months.

## 4. Discussion

The successful use of an off-the-shelf bioengineered conduit in CABG represents a significant advancement in coronary revascularization. Traditional autologous grafts, such as saphenous vein and radial artery, remain the most commonly used conduits but are limited by donor site morbidity, variable patency, and lack of availability in patients with prior vein harvesting or vascular disease. These limitations often necessitate alternative strategies, particularly in high-risk or complex cases [2,3].

While this patient lacked suitable venous or radial arterial conduit, it is important to contextualize TEV performance against all available conduit options, including venous and arterial grafts. The radial artery, increasingly favored as a second arterial conduit, offers superior patency compared to SVG at 5 and 10 years [5]. However, radial artery use is constrained by anatomical availability, the need for preoperative assessment, harvest site complications including hand ischemia and neuropathy, and unsuitability in patients with peripheral vascular disease or Raynaud’s phenomenon. In the present case, the patient had no suitable venous or radial conduit available. The TEV may be particularly advantageous for patients in whom both venous and arterial conduits are unavailable or of poor quality, a clinical scenario of increasing frequency in the aging CABG population. Importantly, unlike venous or arterial conduits, the TEV does not require harvesting from the patient, eliminating donor site morbidity entirely.

In this first European clinical case, the Vascudyne TEV conduit with ESS demonstrated excellent intraoperative handling, showing the TEV anastomosis time to be equivalent to the standard autologous vessel, and favorable short-term postoperative outcomes, including high flow rates at implant and confirmed patency at one month. Transit-time flow measurement is a validated intraoperative quality control tool that predicts early graft failure when values fall outside accepted thresholds. A pulsatility index below 5.0 is generally accepted as indicative of a technically satisfactory coronary bypass graft [6]. The TEV demonstrated a flow rate of 110 mL/min with a PI of 1.0, both of which compare favorably not only to the threshold for adequacy but also to the concurrent LIMA graft (80 mL/min, PI 3.5). The higher absolute flow rate in the TEV likely reflects the larger target vessel caliber (RCA vs. LAD) and the absence of competitive native flow, while the low PI suggests minimal resistance throughout the conduit length—consistent with an absence of kinking, anastomotic narrowing, or distal runoff limitations. These intraoperative hemodynamic parameters, while not predictive of long-term patency, provide important early evidence that the TEV functions as a low-resistance conduit under physiologic coronary hemodynamics. Size mismatch between the 4 mm TEV and the native RCA target vessel, estimated at approximately 2 mm at the distal anastomosis site, was managed using standard surgical techniques. The distal anastomosis was constructed using a beveled end-to-side technique with 7-0 Corolene suture, which allows the surgeon to tailor the anastomotic orifice to the recipient vessel diameter. This approach is identical to the technique routinely used when grafting the saphenous vein (typically 3.5–5 mm) to coronary targets and does not require special adaptation for the TEV. The compliance of the TEV wall permitted gentle molding at the anastomotic site. Proximally, the 4 mm TEV was anastomosed to the ascending aorta using a standard aortotomy and 6-0 Corolene suture, where the size difference is of no hemodynamic consequence given the large caliber of the aorta. Transit-time flow measurements confirmed the absence of significant resistance or turbulence at either anastomosis.

The TEV design, a decellularized human extracellular matrix that supports recellularization, addresses key challenges associated with synthetic grafts, such as thrombosis, immune response and poor integration, while eliminating the need for autologous vessel harvest [11,12]. A notable feature of the TEV is that it does not require immunosuppressive therapy. The decellularization process removes all cellular components, including cell-surface antigens (e.g., MHC class I and II molecules) that would normally trigger an adaptive immune response in the recipient. Residual DNA content is reduced to below immunogenic thresholds, and MHC-I ELISA measurements confirm negligible antigen burden in the final product. This absence of immunosuppression requirement represents a significant clinical advantage over other cell-based or xenogeneic tissue-engineered approaches and simplifies the postoperative management protocol. The regenerative potential of this tissue-engineered product is supported by an established preclinical foundation [12,13]. The decellularization process removes cellular components while preserving the extracellular matrix architecture, which provides a scaffold conducive to host cell infiltration and endothelialization. This was demonstrated by the recellularization and the development of a functional neointima in a baboon arteriovenous graft model, providing mechanistic evidence that the TEV can integrate as a living conduit rather than functioning as a permanent prosthesis [12]. This regenerative behavior distinguishes the TEV from conventional synthetic grafts, which are associated with higher thrombosis rates in small-diameter configurations and cannot remodel in response to hemodynamic stimuli [11].

The rationale for adding oral anticoagulation to aspirin reflects the unique biological characteristics of the TEV at the time of implantation. Unlike autologous vein or arterial grafts, which possess an intact endothelium at the time of surgery, the TEV is implanted as an acellular scaffold without a functional endothelial layer. Until host-mediated endothelialization occurs, a process demonstrated to begin within the first weeks to months in preclinical models, the luminal surface may be more thrombogenic than a native vessel. Dual antithrombotic therapy (antiplatelet + anticoagulant) was therefore selected to provide additional protection during this vulnerable pre-endothelialization window. Apixaban, a direct oral factor Xa inhibitor, was selected over vitamin K antagonists (e.g., warfarin) based on several considerations: (1) more predictable pharmacokinetics without the need for INR monitoring, (2) a favorable bleeding profile relative to warfarin, and (3) rapid onset of action, allowing earlier therapeutic anticoagulation postoperatively. Among NOACs, apixaban has demonstrated the most favorable safety profile with respect to major bleeding in large randomized trials (ARISTOTLE, AVERROES). While no clinical trial data exist specifically for NOAC use with tissue-engineered grafts, the COMPASS trial [21] demonstrated that low-dose rivaroxaban combined with aspirin reduced major cardiovascular events in patients with stable atherosclerotic disease, providing a conceptual rationale for combined antiplatelet-anticoagulant therapy in the post-CABG setting. The duration and intensity of anticoagulation following TEV implantation remains an important question for future clinical investigation and may ultimately be tailored to imaging or biomarker evidence of graft endothelialization.

The use of an external stent support system in this case is an important technical consideration. External stenting of saphenous vein grafts has been evaluated as a strategy to reduce perioperative graft distension injury and limit intimal hyperplasia. The VEST trial used the external support to reduce intimal hyperplasia in saphenous vein grafts [4]. For the TEV, external support serves a mechanical support role: providing the conduit with kink resistance in the dynamically moving environment of the heart. This combination of biological regeneration and mechanical reinforcement represents a novel strategy for achieving durable coronary revascularization.

Several limitations of this report must be acknowledged. The single-patient design and the 1-month follow-up period are significant limitations of this report. While confirmed patency at 30 days excludes early technical failure and acute thrombosis, the critical period for graft failure driven by intimal hyperplasia, accelerated atherosclerosis, and compliance mismatch typically manifests at 6–12 months, the period at which saphenous vein graft failure rates rise markedly [6,7]. The planned 6-month computed tomography coronary angiography and 12-month invasive angiographic follow-up will provide critical data on intermediate-term patency. Furthermore, this case was performed under a specific clinical trial protocol in a carefully selected patient, and broader generalizability will require enrolment of larger, more heterogeneous cohorts. Larger, prospective, multi-center trials will be required to establish long-term safety and efficacy. Several technological developments are anticipated as this platform advances through clinical evaluation. Manufacturing scalability under GMP conditions and optimizing the postoperative pharmacological regimen, particularly the transition from anticoagulation to antiplatelet-only therapy as endothelialization progresses, will be a key focus of future clinical studies.

Nevertheless, the first European clinical implantation of an acellular tissue-engineered vessel for coronary artery bypass grafting provides evidence that complements the existing preclinical and peripheral vascular clinical datasets. Should efficacy be confirmed in further studies, bioengineered conduits like the Vascudyne TEV could transform coronary artery bypass grafting practice by expanding treatment options for patients without suitable autologous grafts, reducing operative complexity, and minimizing complications associated with autologous vessel harvesting [8,9,10]. Conceptually, the TEV represents a paradigm shift from passive conduit replacement to active biological integration. Unlike synthetic grafts (e.g., ePTFE, Dacron), which function as permanent prostheses with well-documented limitations in small-diameter coronary applications, the TEV is designed to serve as a scaffold that transitions into a living vessel through host-mediated recellularization. Preclinical evidence from the 18-month ovine CABG model (unpublished) demonstrated endothelialization, smooth muscle repopulation, and sustained patency following withdrawal of anticoagulation, all hallmarks of biological integration rather than passive material tolerance. If this regenerative trajectory is confirmed in human coronary implants, the implications extend beyond conduit substitution. An off-the-shelf, regenerative conduit could enable surgical revascularization in populations currently excluded from CABG, reduce the dependence on autologous harvest, and potentially eliminate the limitations of native conduits.

## 5. Conclusions

This case report describes the first successful clinical implantation of Vascudyne’s off-the-shelf acellular tissue-engineered vessel for coronary artery bypass grafting in Europe, establishing proof of concept for bioengineered coronary revascularization. The TEV demonstrated surgical handling equivalent to the autologous conduit, intraoperative hemodynamics with accepted performance thresholds (flow rate 110 mL/min, PI 1.0), and confirmed patency at one month. For patients without suitable autologous grafts, a population of growing clinical importance, the TEV combined with external support structure offers a viable conduit alternative that eliminates harvest site morbidity while providing a scaffold for biological regeneration. Prospective, multi-center trials with extended follow-up are now needed to establish the long-term safety, durability, and regenerative performance of this technology in the coronary circulation.

## Figures and Tables

**Table 1:** Patient baseline characteristics.

Characteristic	Value
Age (years)	73
Gender	Male
Height (cm)	165
Weight (kg)	88.4
Smoker Status	Never Smoker
Diabetes	Yes, treated with oral antihyperglycemic medication
Hypertension	Yes, controlled by medication
Hyperlipidemia	Yes, controlled by medication
COPD	No
NYHA Class	I
LVEF (%)	59
Creatinine (mg/dL)	1.24

**Table 2:** Flow measurements of LIMA and TEV grafts after implant and Protamine.

Graft	Flow Rate (mL/min)	Pulsatility Index (PI)
LIMA	80	3.5
TEV	110	1.0

## Data Availability

The original contributions presented in this study are included in the article. Further inquiries can be directed to the corresponding author.
